# National Childhood Diabetes Program Activities in Turkey

**DOI:** 10.4274/jcrpe.1797

**Published:** 2015-03-05

**Authors:** Şükrü Hatun

**Affiliations:** 1 Kocaeli University Faculty of Medicine, Department of Pediatric Endocrinology and Diabetes, Kocaeli, Turkey

**Keywords:** Diabetes, childhood program, Turkey

## Abstract

Recent census figures in Turkey show that out of a population of 76.6 million, 22.7 million (29.7%) are younger than 18 years old. The great majority (>95%) of pediatric cases of diabetes in Turkey are type 1 diabetes mellitus (T1DM). In recent years, with increase in number of pediatric endocrine centers around the country, the important issue of care for diabetic children and adolescents has been revived and major steps have been taken for improvement in pediatric care and its outreach to all diabetic children. The Childhood Diabetes Group continues its activities in cooperation with the Turkish Ministry of Health. A list of areas of interest of the Group include “School programs”, “Incidence/prevalence studies and national registry system”, “Educational guidelines for diabetes in children”, “Increasing the numbers of camps and summer schools for diabetic children”, “Organization of educational programs for the health team”, “National guidelines for transition of diabetic children to adult clinics”, “Improvement of school canteens”, “Educational spots” to improve awareness of diabetes. The activities of the Childhood Diabetes Group will be discussed in detail in this article.

## INTRODUCTION

Recent census figures in Turkey show that out of a population of 76.6 million, 22.7 million (29.7%) are younger than 18 years old. The great majority (>95%) of pediatric cases of diabetes in Turkey are type 1 diabetes mellitus (T1DM). However, reports of T2DM are noted to be on the increase in recent years.

Programmed activities relating to improvement in diabetes care in Turkey have been initiated in 1994 (1). One of the high points of the first meeting organized by the Ministry of Health was the decision to organize a separate section for pediatric and adolescent diabetes. Accordingly, a committee consisting of Ministry of Health personnel and pediatric endocrinologists was formed; this “Pediatric and Adolescent Diabetes Group” met in April 1995 and planned to undertake their activities under three headings, namely: 1) Data collection and epidemiology, 2) Education and 3) Quality improvement. Especially the data collection activities of this group were programmed and started immediately and lasted one whole year (1). Unfortunately, after this first year, the group dissolved due to lack of motivation amongst its members to continue this work and lack of adequate support.

In recent years, with increase in number of pediatric endocrine centers around the country, the important issue of care for diabetic children and adolescents has been revived and major steps have been taken for improvement in pediatric care and its outreach to all diabetic children. The accomplishment of these pediatric endocrinology centers and the courses for health workers organized yearly since 2001 have played an important role in progress of diabetic care. At the first meeting of the Consultants’ Group for Control of Diabetes in Turkey, organized by the Turkish Ministry of Health aiming to improve the ongoing activities relating to diabetes, care for diabetic children has been discussed as a major issue and a decision has been taken to consider “Improvement of Care and Treatment of Diabetic Children” under a separate heading. This important decision has been relayed to the members of the Pediatric Endocrinology and Diabetes Society and a work group on diabetes has been started amongst the members of this organization. The first meeting of this work group was held on October 3, 2009 in Ankara and was attended by a large group pediatric endocrinologists. Problems related to pediatric diabetic care were discussed in depth at this meeting and a decision to work in subgroups was taken. Thus, separate working groups for separate issues such as “epidemiology”, “teamwork in diabetes care”, “education and awareness”, “T2DM and risk factors”, were instituted. Also at this meeting, a decision was taken to strengthen the impact of planned activities by inclusion of nurses, dietitians and workers in other related branches in the activities related to pediatric diabetes care through their participation. An evaluation of the work accomplished to date in diabetes care for children in Turkey is presented below.

### Present Issues

1- A national registry for children with T1DM has not yet been established in Turkey. For this reason, detailed epidemiological data relating to T1DM are not available. However, based on the data of the National Labor Institution, results of a study on nationwide figures for incidence and prevalence of diabetes in individuals under 18 years of age, are available (2). According to this study, the incidence of T1DM in Turkey in the population under 18 years is 10.8/100 000 and there are 17 175 children (prevalence 0.75/1000) with T1DM who are presently being followed and given care. In another recent study on the prevalence of T1DM among 6-18 years old children, a prevalence figure of 0.66/1000 was reported (3). According to data obtained from the patient files of the Hacettepe Children’s Hospital in Ankara, comparing the figures between 1991 and 2010, age of onset of T1DM has shown a decrease in recent years, from a mean age of 9.5 years to age 7.0 years (4). On the other hand, the records of the Marmara University Pediatric Endocrinology Center indicate an increase in age of onset (age 7.25 years in the years 1999-2003; age 9.0 years in the time period between 2009-2013) (5). There is a need for further more extensive nationwide data on this issue.

2- A major section of the community, including teachers and at times health personnel, define diabetes as a disease limited to adults. Occasionally, even when a pediatric patient exhibits striking symptoms of diabetes, a diagnosis of T1DM may be missed. For this reason, diagnosis is often delayed and rate of presentation with diabetic ketoacidosis (DKA) is higher compared to many western countries. A recent nationwide study from 33 centers on 405 T1DM pediatric patients followed long term reported a frequency of DKA at diagnosis as 45.7% (severe DKA with a pH value of <7.1 in 13.3%) (6).

3- At present, social security regulations in Turkey cover the population under age 18 years unconditionally. This is a major advantage. A large proportion of children with T1DM are followed by the Pediatric Diabetes Teams, under the leadership of a pediatric endocrinologist or a specialist in pediatric diabetes. There exist 50 pediatric endocrinology centers around the country and about 50 nurses and 50 dietitians work in these centers. Each year, 7 camps for diabetic children are organized by pediatric centers in various parts of Turkey (İstanbul University Faculty of Medicine, Kocaeli University Faculty of Medicine, Aegean University Faculty of Medicine, Dokuz Eylül University Faculty of Medicine and Faculties of Medicine in Antalya, Diyarbakır and Samsun). Many of the centers use a flexible regimen in following their patients. Despite these advances, a recent study has shown that the mean hemoglobin A1c (HbA1c) level of the diabetic children followed by these teams was 8.5%. Two thirds of these patients had HbA1c levels higher than 7.5% (7). In another study, 33% of patients were found to have HbA1c levels >9% and the proportion of patients showing a poor control was higher in the European part of the country (8). Data on rate of microvascular complications are limited.

4- Although the number and geographic distribution of pediatric endocrinology centers appear to be adequate, not all children with T1DM are followed in these centers. Another point is the lack of an adequate number of staff (1 nurse for 100 T1DM patients, 1 dietitian for 200 T1DM patients) in the pediatric endocrinology centers. The foremost reason for this is the lack of officially accepted norms for staffing these centers with nurses, dietitians and psychologists who are trained in pediatric diabetes. Another issue that requires attention is the responsibility of the school system in the care of children with T1DM.

5- Although many groups have strived for the education of the children with T1DM over the years, a structured educational programme reaching all T1DM patients has not yet been put into effect. The number of camps for diabetic children is also still small. There is also a need for updating the existing educational materials and adapting them for online use.

6- There has been important advances in the Turkish social security system in recent years which have served to improve the care of diabetic children. However, the system still fails to meet the full cost of new technology.

7- In Turkey, obesity in children is increasing at a fast rate. As a consequence, an increase is also seen in the rate of cases with metabolic syndrome. Cases of T2DM have also been reported more frequently in recent years.

### Studies Aiming to Develop a National Program for Diabetic Children and the 2015-2020 Program

Within the framework of the present situation and needs summarized above, the Pediatric Diabetes Working Group had a second meeting in İstanbul on 16-17 January 2010 and discussed the solutions to the existing problems. Also, the Work Group on the Strategy and Control of Diabetes of the Turkish Ministry of Health met in Ankara on 15-20 February and at this meeting the previous decision of accepting Diabetes in Children under a separate heading was confirmed to be a definite part of the national diabetes programme. During this same meeting, a tentative programme focusing on “Problems and Targets” was designed and this programme took its final form on 4 March 2010 at a meeting organized by the Ministry of Health under the heading “The second meeting of the Work Group on the Diabetes Control Program for Turkey” and was held in Ankara (9). Four strategies were discussed and accepted at this meeting:

1. Steps to increase awareness and knowledge on the characteristics and treatment of diabetes in children,

2. Steps to prevent prejudices and misinformation about diabetes in children,

3. Steps to provide social support to enable diabetic children to live a healthy life and to make a healthy transition to adulthood, 

4. Steps to improve the care and treatment offered to diabetic children throughout the country, hence to improve metabolic control. 

These strategies have been amplified by the Work Group of the Pediatric Endocrinology and Diabetes Society. The objectives and details of activities foreseen for the realization of the objectives relating to diabetes care in children were stated as below: 

1- To take steps for establishment of a nationwide recording system to cover type 1 and type 2 diabetic patients of ages 0-18 years. 

a. To realize this recording system by its incorporation into the family physicians’ recording system and to modify the entry details in accordance with changing needs,

b. To enable the data pertaining to the pediatric and adolescent diabetics under a separate filing system, thus to be able to follow the changes in the epidemiology of T1DM over time, 

c. To run cross-sectional prevalence surveys at 5 years intervals on pediatric and adolescent diabetes, covering the whole country, using a uniform methodology.

2- To take measures to enable early diagnosis and thus reduce the frequency of presentation with DKA. 

a. To increase awareness of T1DM in the community, to prepare educational materials and spots on the signs and symptoms of T1DM and of DKA for the public. 

b. To continue the school programmes on diabetes including efforts on increasing awareness in the teaching staff. 

c. To make efforts for inclusion of T1DM in educational programmes targeting family physicians and pediatricians and to support similar programmes in all regions.

d. To make efforts for inclusion of clinical findings of T1DM in children as a topic in educational programs for health personnel working in emergency settings.

e. To make efforts for inclusion of a short chapter on “Diabetes in children” in the curricula of primary schools.

f. To make efforts to convince the Turkish Ministry of Education to recognize 14 November, the World Diabetes Day, as a day for “Awareness of Diabetes” and using this occasion, to popularize this subject in all schools through educational meetings and educational materials addressing teachers and students of different age groups.

3- To develop national standards for the care and treatment of diabetic children.

a. To assess the deficiencies of the clinics offering outpatient care to diabetic children regarding their human workforce and their basic requirements and to make efforts to overcome these deficiencies. 

b. To continue supporting school programs for diabetes, to make efforts to improve the school life of diabetic children, to support educational sessions for the school administrators and teachers.

c. To include a clause on “visiting the local schools attended by diabetic children and offering a structured educational programme for the teachers in these schools” in the listing of the tasks of nurses working in community centers. 

d. To initiate a certificate course for nurses on “Pediatric Diabetes”.

e. To make efforts to increase the number of teams who work in camps for diabetic children, to procure the means of the Social Security Agency in meeting the costs of patient participation to these camps, to organize day camp programs held on holidays, to prepare a guidelines book for diagnosis and treatment of diabetes in childhood and adolescence and to develop an algorithm for referral.

f. To make efforts for preparation of national guidelines and forms by a joint team of adult and pediatric endocrinologists for use in the transition of the pediatric cases to adult clinics. 

g. To make efforts to meet the special needs of diabetic patients attending entrance examinations to schools and universities and to obtain the cooperation of the Ministry of Education and of the Ministry of Health in the preparation of a relevant guideline.

4- To realize the outreach of standard/adequate education to all diabetic children and their families.

a. To make efforts for provision of supplementary resources for diabetes education through changes in bylaws. 

b. To promote the use of Guidelines for Educators in Diabetes in Childhood in the education of diabetic children and their parents.

c. To prepare a basic educational book based on diagrams and illustrations for diabetic children and their parents. To also prepare this text for online reach.

d. To prepare an educational videogame and present it at our website http://diyabet.gov.tr. 

5- To take steps to prevent T2DM in children and also to prevent childhood obesity which is the most important risk factor for T2DM in adults.

a. To make efforts for inclusion of subjects such as nutrition, obesity and diabetes in the curricula of teachers colleges. 

b. To make efforts for inclusion of childhood obesity as a separate module within the Turkish program “Healthy nutrition and a physically active life (2014-2017)”. 

c. To organize a national campaign aiming to reduce time spent by the children watching TV.

d. To make efforts for inclusion of “childhood obesity and healthy nutrition” among the science chapters of primary school textbooks.

e. To widen the scope of the existing list of items which are legally allowed to be sold in school food shops. 

6- To enlarge the scope of social insurance for children with T1DM so that it covers all their needs for treatment and follow-up; to cover the social security needs of these patients throughout their lifetime. This insurance should cover:

a. The items for measurement of ketones in blood and urine.

b. The insulin pumps and their sets and all equipment and drugs needed in the long-term treatment of T1DM patients.

c. To make efforts for realization of legal measures to provide insurance to those patients who are older than age 18 and who are not covered. 

d. Provision of education and adequate nutrition to diabetic children from needy families.

### Present Activities and Areas of Interest of the “Pediatric and Adolescent Diabetes Group”

The Group continues its activities in cooperation with the Turkish Ministry of Health. A list of areas of interest of the Group include “School programs”, “Incidence/prevalence studies and national registry system”, “Educational guidelines for diabetes in children”, “Increasing the numbers of camps and summer schools for diabetic children”, “Organization of educational programs for the health team”, “National guidelines for transition of diabetic children to adult clinics”, “Improvement of school canteens”, “Educational spots to improve awareness of diabetes”. Of these headings, the activities of the Group on “School programs” and “National guidelines for transition of diabetic children to adult clinics” will be discussed in detail below.

### School Programs for Diabetic Children in Turkey

There are approximately 20 000 children, mostly of school age, living with diabetes (mostly type 1) in Turkey. Unfortunately, many children living with T1DM and their families encounter problems in accessing an optimal school environment and in participating in school programs. According to a recent study, upon returning to school, the HbA1c of children with T1DM increased and they had problems with their diabetes care. Some children with T1DM are forbidden to participate in physical education and some activities such as school trips are off-limits. It has been reported that some infant nurseries and kindergartens refuse registering children diagnosed with diabetes to avoid the responsibility of care. Although it is well known that diabetic children should not only be allowed access but also be motivated to participate in all activities with their schoolmates, it is also known that, due to little awareness about diabetes, these children are sometimes labeled as sick and are held exempt from many activities. To accommodate students with diabetes, a school nurse or a counselor should be trained on basic diabetes care. Other diabetes care requirements include an allotted space for measuring blood glucose and injecting insulin, provision of healthy school meals and toilet breaks during classes. Moreover, teachers should learn the signs and symptoms of diabetes and also be able to recognize the early signs of DKA and thus prevent development of serious events.

The issue of diabetes care and detection concern in schools has become even more complex with the growing problem of weight gain and obesity in childhood. Obesity frequency among the age group of 6 to 16 years has increased from 5% to 10.5% (16.3% among the high income level group) in the last eight years in Turkey. At least one third of these children are also at increased risk for adult obesity and/or T2DM. Childhood obesity is caused primarily by poor lifestyle habits which include consumption of high-calorie or processed junk food such as sugar-sweetened beverages and lack of physical activity. Preventing obesity in adulthood depends mainly on the prevention efforts realized in childhood and around puberty. In recent years, the Turkish government has paid more attention to childhood nutrition and the Radio and Television Supreme Council has put limitations on advertisements that promote high-calorie foods. The Ministry of Education has also restricted the sale of junk food and sugar-sweetened beverages in schools.

The Diabetes Program at School, initiated by our Diabetes Working Group in 2010, was developed in cooperation with the Ministries of Education and of Health as a joint protocol. Target groups of the Diabetes Program at School include all professional teachers across Turkey including teachers who currently oversee the education of children with diabetes, members of the pediatric diabetes medical team, local representatives of the Ministries of Education and Health and school administrators.

The objectives of the program can be listed as: 1) To raise school community and teacher awareness about T1DM, 2) To promote early diagnosis of T1DM and decrease the frequency of DKA among school children, 3) To promote provision of better care for children with diabetes, 4) To create a healthy nutrition attitude among school children, 5) To increase awareness about obesity.

The Diabetes Program at School succeeded in drawing together a large group of teachers and securing broad coverage from the national press. Almost all teachers who participated in the national meeting went on to organize diabetes meetings in their respective schools with the assistance of local authorities. In 2011, a one-day training meeting was organized across Turkey, which included teachers, government officials and pediatricians involved in diabetes and in obesity. By World Diabetes Day 2011, the entire campaign was launched and millions of school children and teachers joined the school training sessions that were organized within the scope of the program. Health workers in diabetes served as facilitators in these sessions. Awareness was further reinforced with a poster campaign, entitled “Does my child have diabetes?” and distributed to 60 000 schools across Turkey, reaching approximately 650 000 teachers and 16 million school children at primary and middle schools. Two brochures on diabetes and obesity in childhood, a guide on diabetes care of the child at school and a training presentation for teachers were also provided. The Ministry of Education sent an official letter to provincial directorates about the Diabetes Program at School and pediatric endocrinology clinics across the country contributed to the outreach of a campaign directed to teachers and school administrators about caring for a child with diabetes in the school environment. Visual materials were utilized as a major component of instruction for the Diabetes Program at School and four short films covering topics such as diabetes care management, prevention of childhood obesity and voices of children living with diabetes were prepared and utilized. A program website offering educational materials and other forms of media was launched (www.okuldadiyabet.org) to expand information and awareness across all of Turkey.

By the end of 2011, over 7.5 million students and nearly 600 000 teachers were trained in 25 thousand schools by a variety of diabetes healthcare experts. Since the first training experiences in 2010, a large number of additional students and teachers joined the training activities in the schools. In 2012, “Teacher Achievement Awards” were established and given to seven teachers, each from a different region of Turkey. Program effectiveness evaluations were done before and after training to the teacher participants. After one initial survey, 85% of the surveyed teachers expressed satisfaction with the diabetes education they received, 89% noted that the program was informative about the symptoms of diabetes and in another surveyed group, 99% of teachers found the website useful for retrieving relevant information. The results of the survey showed that the online education platform and website helped teachers understand the symptoms of diabetes and led to increased awareness for early diagnosis. The teachers also reported greater understanding of the healthcare needs of students with diabetes at school and the importance of healthy eating, such as avoiding junk food at school. To the credit of the program, 30 teachers who had undergone diabetes training succeeded in the early diagnosis of diabetes in 40 children.

### Transition of Diabetic Children to Adult Clinics

The transition period from childhood to adulthood covers the time period between ages 18 to 30 years. In the early years of this period (18 to 24 years), many diabetic children suffer from feelings of separation/being cast away from the environment they are used to. They feel squeezed between their geographic and economic state, their feelings and their diabetic state, hence they feel low and lack self-confidence. Diabetic children and adolescents in this age group, like their peers, are still immature. They feel that they are not vulnerable and thus cannot be harmed, they underestimate risk situations, revolt to authority, feel at odds with the members of their family and refuse to share their troubles with them. These adolescent traits, in a diabetic child, lead to loss of metabolic control, to delays in transition, to problems in attendance to the clinic, to neglect in following the treatment program and consequently, to an increase in hospitalization and increase in acute complications and deaths.

In the transition period, the diabetic children are faced on the one hand with a necessity to adapt to a change from a family-centered model based on dependency to one where they need to be independent and at the same time adapt to the crowded, T2DM-focused environment, to adult dialogues and to the care which is more “routine”. Consequently, many suffer from difficulties in communication (10). In addition, at this stage of life, they are faced with problems related to professional/academic success, with problems in their attempt to establish their personality, with problems related to military service, romantic relationships and marriage. All these make this transition period a difficult one and one that most diabetics remember as a difficult period in their life.

For all the above reasons, it is most important to keep the patient “in good metabolic control” during this transition period and to this end, a cooperation between the pediatric and adults centers becomes necessary. The most important points in dealing with diabetic children during this transition period is not to leave the family and the child alone in this difficult period, to ensure that the transition is well designed and to start the preparations for transition well ahead of time. These preparations need to include organization of sessions on communication attended by pediatric and adult physicians and also by the patients and their families. A detailed information sheet on each patient should be presented at these meetings.

All the problems listed above are also relevant for diabetic children in Turkey. An important section of these children, when they reach pubertal ages (14 years and above), are noted to neglect attendance to their routine follow-up. Particularly at ages of transition to adult clinics (18 to 21 years), they are noted to be disinterested in their diabetic state. Thus patients in this age period, a time period which requires meticulous care for many diabetics, are exposed to the risks of developing the acute and/or chronic complications of diabetes.

For the above reasons, also taking into consideration the recommendations of the American Diabetes Association Working Group (11) and the points emphasized in the IDF/ISPAD Guidelines (12) (recently translated into Turkish, awaiting publication), a “Guideline for Transition of Diabetic Children to Adult Diabetes Clinics” has been prepared by a work group consisting of pediatric and adult endocrinologists, nurses, dietitians, psychologists and young adult diabetic patients. The main points mentioned in this guideline are listed below:

1. Recommendations for Pediatric Endocrine and Diabetes Centers Relating to Transition 

a. Appoint one of the nurses in your Center as the “Guide for Transition” 

b. Start the preparatory work for transition one year before the date of the transition. 

c. Review the knowledge and self-care potentials of the diabetic patient, provide the necessary support. 

d. Prepare a detailed file on the clinical data pertaining to the patient. This file should include information on metabolic control, problems and complications, treatment details, patient’s level of self-care, medical history, family and social history and social security data. The Guideline includes a model for this file. 

e. Depending on the number of patients which have reached the age for transition, organize 2-4 sessions per year for transition of patients to the adult clinic. The maximum number of patients discussed at each session should not exceed 10. Make sure that the persons appointed as “guides for transition” in the pediatric and adult centers and the pediatric and adult specialists attend these sessions. In the first part of these sessions, information is given by the pediatric team to the adult team and subsequently, the patient and his/her family are introduced to the adult team. 

f. If your patient has some specific problems pertaining to social security or other items, make a note of these problems in a separate file. 

g. Discuss the importance of continuous uninterrupted follow-up and care for a diabetic patient and the risks for development of complications if this care is neglected.

h. In the transition process, the conditions of the patient and of the family need to be taken into consideration. The patient or his/her family cannot be forced for transition to the adult center within the same hospital. In such cases, the patient and his/her family need to be informed on the need to be followed by an experience and qualified team. 

i. Patients who are to be transferred to an adult clinic which does not have a specialist in diabetes need to be followed and contact must be made with the responsible clinician. 

j. Continue to follow the patients whom you have transferred to the adult clinic. Continue to remain in contact with the “Guide for Transition” person at the adult clinic and thus ensure the continuation of care for your patients. 

2. Recommendations for Adult Endocrine and Diabetes Centers Relating to Transition 

a. Appoint one of the nurses in your Center as the “Guide for Transition” and make sure this person remains in contact with the “Guide for Transition” at the Pediatric Center. 

b. Attend the sessions on transition regularly. 

c. Study the “transition file” sent by the pediatric center carefully. If the patient has been sent without the file, make sure to ask for the file from the pediatric center.

d. If possible, have the transferred patient be followed by the same doctor (or consultant) on the same day of the week. 

e. Considering that the diabetic patients who went through the transition are accustomed to the care of the pediatric center and may be in a state of anxiety, spare more time and effort for these patients to get accustomed to this new environment. 

f. If possible, arrange for a separate day for these patients at your outpatient clinic.

g. Arrange for the nurses at your center to attend the camps for diabetic children and to meet the pediatric diabetes teams. 

h. Arrange for separate educational sessions for the patients who have been transferred from the pediatric centers and try to provide occasions for peer support for these patients.
